# Supplementary material for: PTPN21 and Hook3 relieve KIF1C autoinhibition and activate intracellular transport
Source: Nat Commun. 2019 Jun 19;10:2693. doi: 10.1038/s41467-019-10644-9 (PMC6584639; doi:10.1038/s41467-019-10644-9)
Supplement: Supplementary file 1 — Supplementary Information [file 41467_2019_10644_MOESM1_ESM.pdf]

Supplementary Information

PTPN21 and Hook3 relieve KIF1C autoinhibition and activate intracellular transport

Nida Siddiqui, Alexander James Zwetsloot, Alice Bachmann, Daniel Roth, Hamdi Hussain, Jonathan Brandt, Irina Kaverina and Anne Straube

Content:

Supplementary Figure 1: KIF1C is a dimer.

Supplementary Figure 2: Verification of intramolecular crosslinks in KIF1C.

Supplementary Figure 3: Verification of intramolecular crosslinks in KIF1C.

Supplementary Figure 4: PTPN21 FERM domain requires either KIF1C or KIF16 to stimulate podosome formation.

Supplementary Figure 5: Verification of crosslinks between KIF1C and PTPN21 FERM domain.

Supplementary Figure 6: PTPN21 binding requires the KIF1C stalk region.

Supplementary Table 1: DNA oligonucleotides used in this study.

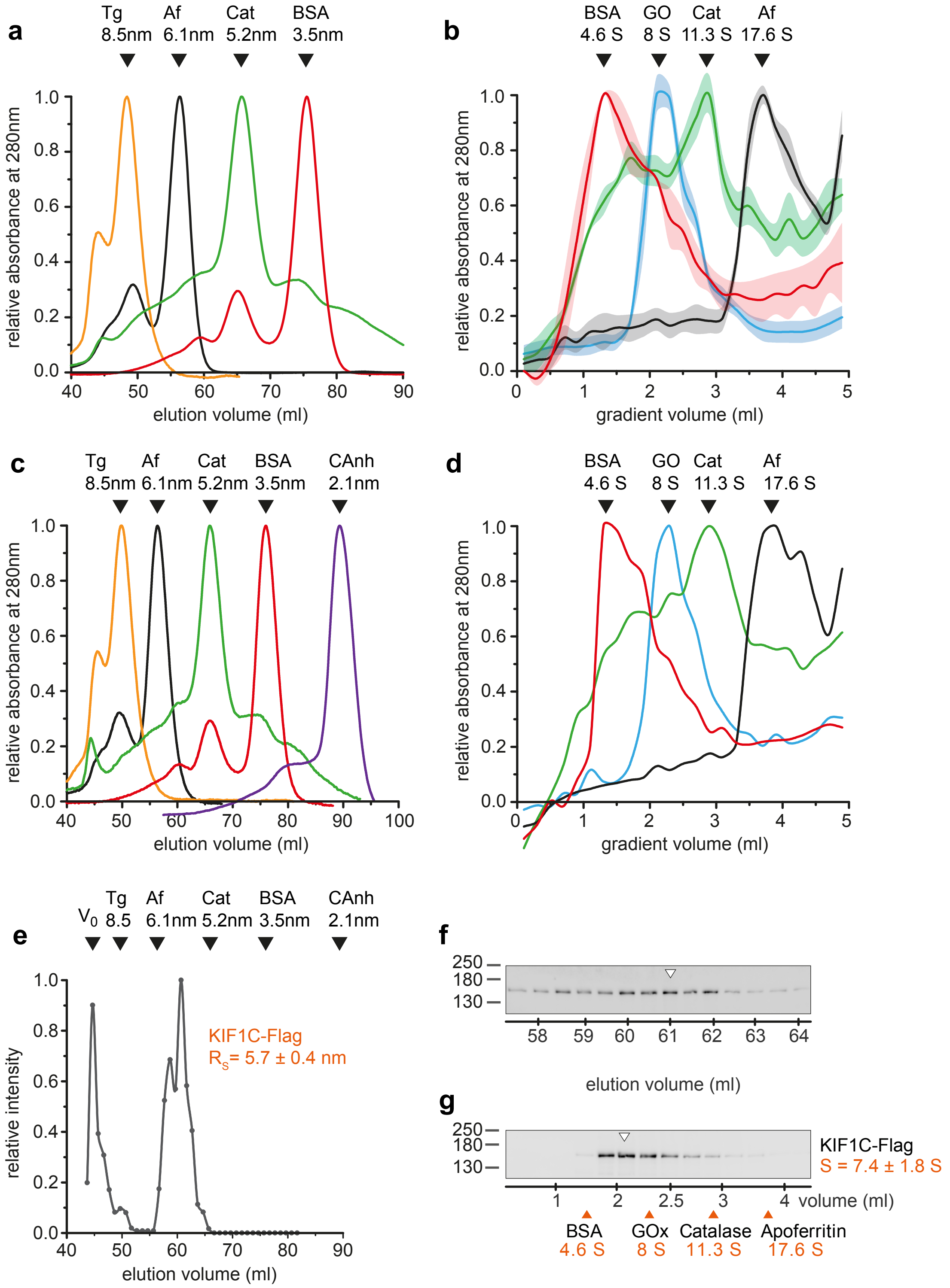

Supplementary Figure 1: KIF1C is a dimer. (a) Representative absorbance traces from size exclusion chromatography of standard proteins (Tg, thyroglobulin; Af, apoferritin; Cat, catalase; BSA, bovine serum albumin) with Stoke's radius as indicated. Related to Figure 1b. **(b)** Absorbance of fractions from glycerol gradients of standard proteins (BSA, bovine serum albumin; GO, glucose oxidase; Cat, catalase; Af, apoferritin) with sedimentation coefficient indicated. Data show Mean ± SEM from 3 independent experiments. Related to Figure 1a. **(c)** Representative absorbance traces from size exclusion chromatography of standard proteins (Tg, thyroglobulin; Af, apoferritin; Cat, catalase; BSA, bovine serum albumin, CAhn, Carbonic Anhydrase) with Stoke's radius as indicated. **(d)** Representative absorbance of fractions from glycerol gradients of standard proteins (BSA, bovine serum albumin; GO, glucose oxidase; Cat, catalase; Af, apoferritin) with sedimentation coefficient indicated. **(e)** Size exclusion chromatography of KIF1C-Flag in HEK293 cell lysates. Elution peaks of standard proteins and void volume V_0 are indicated by arrowheads. Fraction in void is probably KIF1C in complex with other motors and adpater proteins. **(f)** Westerrn blot of KIF1C-Flag peak fractions from (e). **(g)** Western blot of glycerol gradient of KIF1C-Flag in HEK293 cell lysates.

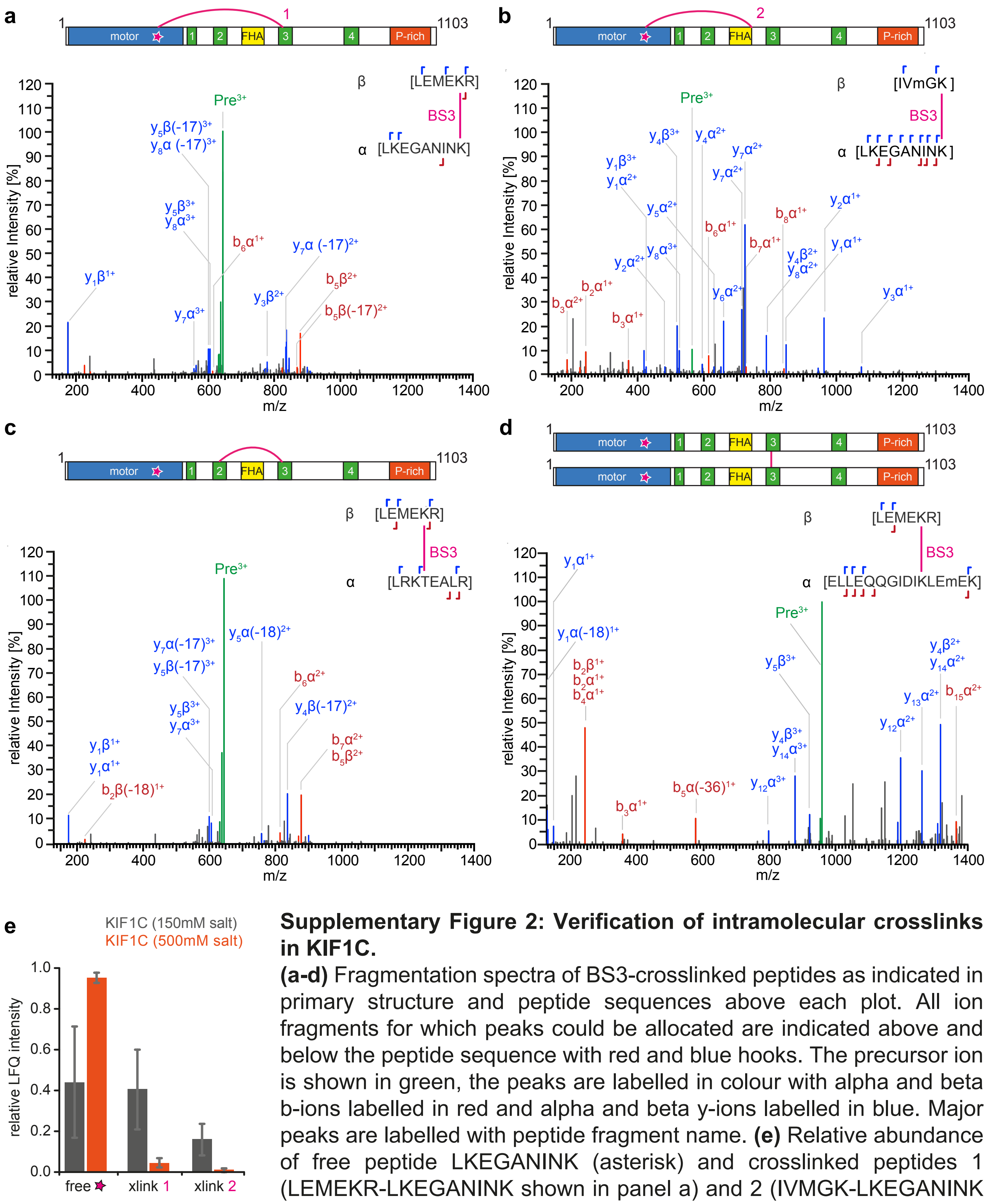

Supplementary Figure 2: Verification of intramolecular crosslinks in KIF1C.
(a-d) Fragmentation spectra of BS3-crosslinked peptides as indicated in primary structure and peptide sequences above each plot. All ion fragments for which peaks could be allocated are indicated above and below the peptide sequence with red and blue hooks. The precursor ion is shown in green, the peaks are labelled in colour with alpha and beta b-ions labelled in red and alpha and beta y-ions labelled in blue. Major peaks are labelled with peptide fragment name. **(e)** Relative abundance of free peptide LKEGANINK (asterisk) and crosslinked peptides 1 (LEMEKR-LKEGANINK shown in panel a) and 2 (IVMGK-LKEGANINK shown in panel b) in samples prepared at physiological salt (150mM) or high salt (500mM). LFQ intensities shown are normalised to summed intensities of all LKEGANINK-containing peptides per sample. n = 3 intdependent experiments. Error bars show SEM.

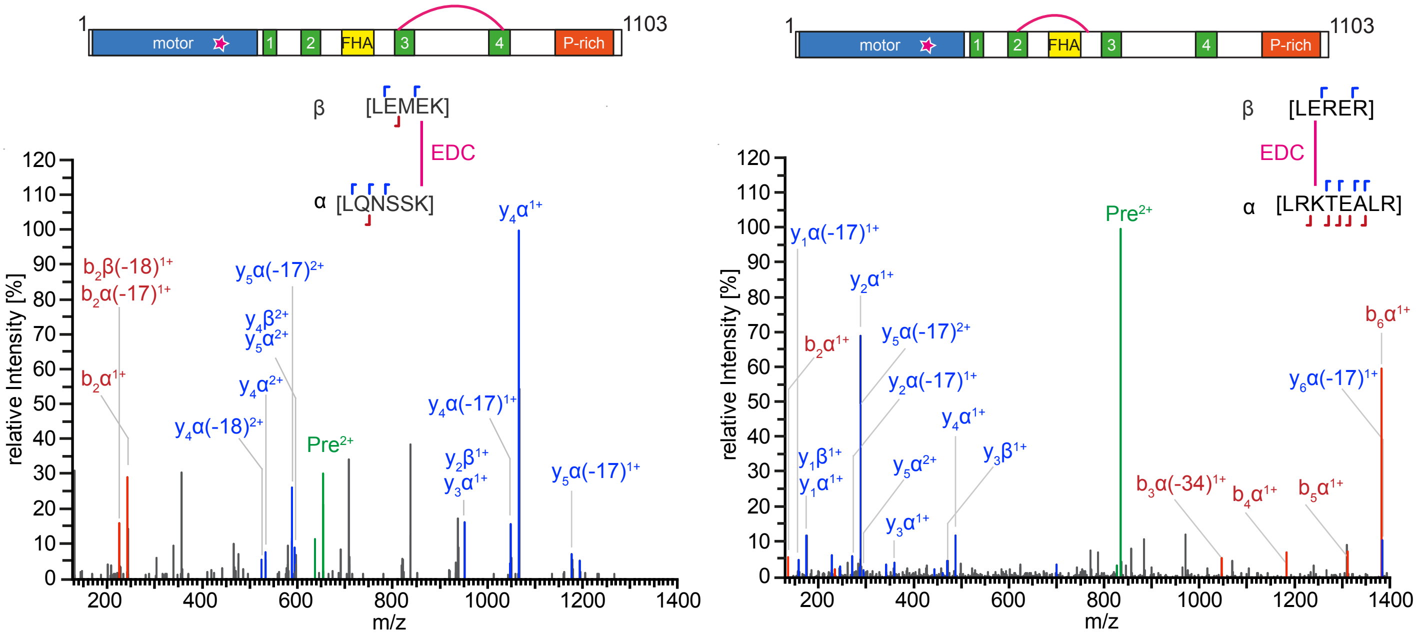

Supplementary Figure 3: Verification of intramolecular crosslinks in KIF1C.
Fragmentation spectra of EDC-crosslinked peptides as indicated in primary structure and peptide sequences above each plot. All ion fragments for which peaks could be allocated are indicated above and below the peptide sequence with red and blue hooks. The precursor ion is shown in green, the peaks are labelled in colour with alpha and beta b-ions labelled in red and alpha and beta y-ions labelled in blue. Major peaks are labelled with peptide fragment name.

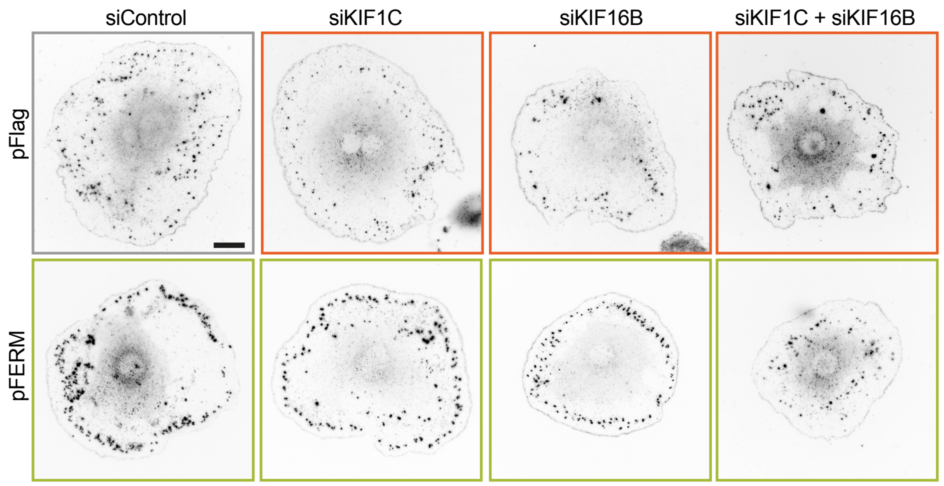

Supplementary Figure 4: PTPN21 FERM domain requires either KIF1C or KIF16 to stimulate podosome formation.
Representative images of podosomes in A7r5 cells treated with siRNAs and rescue plasmids as indicated. Podosome formation was induced by treatment with 5µM PDBu for one hour before immunostaining for cortactin. Scale bar 20µm. See Figure 4d for quantitative data.

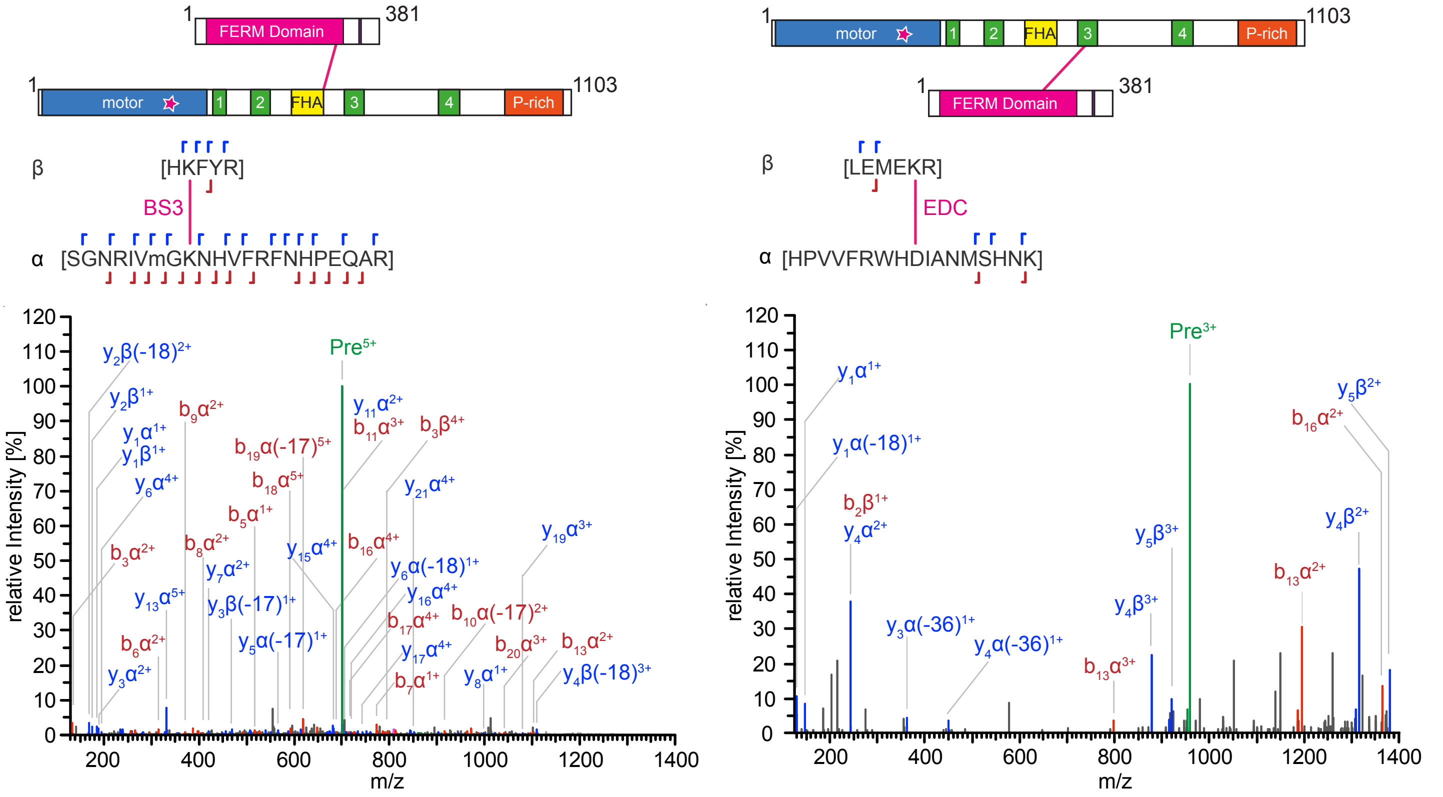

Supplementary Figure 5: Verification of crosslinks between KIF1C and PTPN21 FERM domain. Fragmentation spectra of crosslinked peptides between KIF1C and PTPN21 as indicated in primary structure and peptide sequences above. The crosslinker is specified in magenta. All ion fragments for which peaks could be allocated are indicated above and below the peptide sequence with red and blue hooks. The precursor ion is shown in green, the peaks are labelled in colour with alpha and beta b-ions labelled in red and alpha and beta y-ions labelled in blue. Major peaks are labelled with peptide fragment name.

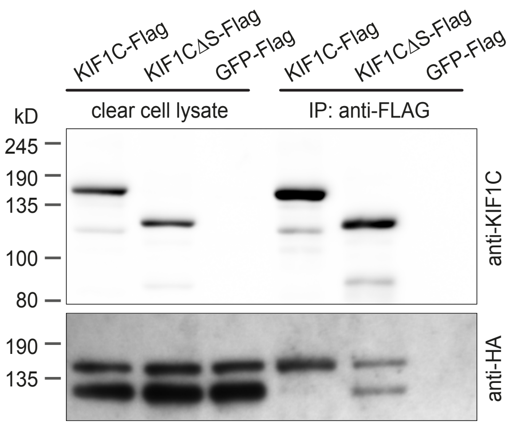

Supplementary Figure 6: PTPN21 binding requires the KIF1C stalk region. Co-immunoprecipitation of HA-PTPN21 with KIF1C-Flag, KIF1CΔS-Flag and GFP-Flag from HEK cell lysates. Note that PTPN21 co-precipitation is reduced when KIF1C stalk is deleted.

Supplementary Table 1: DNA oligonucleotides used in this study

Oligo name	Oligo sequence
AS370 (SalI-KIF1C(679) forward)	5'-CGGGGtcGACTCTGACAAGCGCTCTTG-3'
AS371 (SalI-KIF1C(825) forward)	5'-GGAGGtcGaCCGAGGGGCGGAGGTGG-3'
AS83 (GFP reverse)	5'-GCCGTTTACGTCGCCGTC-3'
UT157 (NdeI-KIF1C forward)	5'-GAcatATGGCTGGTGCCTCGGTG-3'
AS813 (KIF1C(349)-NcoI reverse)	5'-GTGGTGGTccatggCACCTGCGGCCGCGTTCACGC-3'
AS696 (EcoRI-KIF1C(612) forward)	5'-GAGTCgaattcGAAATGGAGAAGAGGCTGCAG-3'
AS778 (KIF1C(922)-NotI reverse)	5'-ATAAATgcggccgcCTACTCCCAGCTTGACAGTGGTG-3'
AS380 (PTPN21 reverse)	5'-GAGCCCTCTGTATTTCTGATG-3'
AS264 (CMV forward)	5'-CGCAAATGGGCGGTAGGCGTG-3'
AS379 (PTPN21 mutagenesis)	5'-GTTGTAGTACCAtAatgaaAAGTAAGTGACC-3'
AS558 (NdeI-HA-tag forward)	5'-AAGCTTcatATGGGATACCCATACGATGTTC-3'
AS592 (PTPN21(381)-NotI reverse)	5'-GAGGTCAgcggccGCTCTATCCAAGCTTGTCTG-3'
AS556 (NdeI-HA-Ezrin forward)	5'-GAAACCcatatgGGATACCCATACGATGTTCCAGATTACGCT GTGGTGCCGAAACCAATCAATGTC-3'
AS557 (Ezrin(328)-NotI reverse)	5'-CGGTTgcggccgcTTTCTTCTCTGTTTCCAGCTG-3'
AS690 (EcoRI-Hook3 forward)	5'-CCGTAgaattcATGTTCAGCGTAGAGTCGCTG-3'
AS691 (Hook3-BamHI reverse)	5'-ACAATAACCGGTggatccCTTGCTGTGGCCGGCTG-3'
AS689 (AscI-Hook3 forward)	5'-ACATAggcgcgccCTATGTTCAGCGTAGAGTCGCTG-3'
