# Supplementary material for: PTPN21 and Hook3 relieve KIF1C autoinhibition and activate intracellular transport
Source: Nat Commun. 2019 Jun 19;10:2693. doi: 10.1038/s41467-019-10644-9 (PMC6584639; doi:10.1038/s41467-019-10644-9)
Supplement: Supplementary file 3 — Description of Additional Supplementary Files [file 41467_2019_10644_MOESM3_ESM.docx]

**Title:** Supplementary Data 1

**Description:** The dataset contains a list of proteins identified in our BioID experiments with full length KIF1C and KIF1CΔS. Weighted spectra outputs are provided for all three independent experiments and any replicates within these. The list is sorted by p-value of the Fisher’s Exact test with significant hits at the top. Proteins are grouped in clusters and rows of cluster members that are not significant hits are hidden. Significant hits are further classified in those that are enriched in either full length or stalk deletion construct samples. Yellow highlighted entries are significant hits enriched more than 10-fold in full length KIF1C over stalk deletion in line with highlighted box in Figure 7b that summarises these data.
