# Supplementary material for: PTPN21 and Hook3 relieve KIF1C autoinhibition and activate intracellular transport
Source: Nat Commun. 2019 Jun 19;10:2693. doi: 10.1038/s41467-019-10644-9 (PMC6584639; doi:10.1038/s41467-019-10644-9)
Supplement: Supplementary file 5 — Reporting Summary [file 41467_2019_10644_MOESM5_ESM.pdf]

nature research

Corresponding author(s): Dr. Anne Straube

Last updated by author(s): May 21, 2019

Reporting Summary

Nature Research wishes to improve the reproducibility of the work that we publish. This form provides structure for consistency and transparency in reporting. For further information on Nature Research policies, see Authors & Referees and the Editorial Policy Checklist.

Statistics

For all statistical analyses, confirm that the following items are present in the figure legend, table legend, main text, or Methods section.

n/a	Confirmed	
☐	☒	The exact sample size (*n*) for each experimental group/condition, given as a discrete number and unit of measurement
☐	☒	A statement on whether measurements were taken from distinct samples or whether the same sample was measured repeatedly
☐	☒	The statistical test(s) used AND whether they are one- or two-sided<br>*Only common tests should be described solely by name; describe more complex techniques in the Methods section.*
☒	☐	A description of all covariates tested
☐	☒	A description of any assumptions or corrections, such as tests of normality and adjustment for multiple comparisons
☐	☒	A full description of the statistical parameters including central tendency (e.g. means) or other basic estimates (e.g. regression coefficient) AND variation (e.g. standard deviation) or associated estimates of uncertainty (e.g. confidence intervals)
☐	☒	For null hypothesis testing, the test statistic (e.g. *F*, *t*, *r*) with confidence intervals, effect sizes, degrees of freedom and *P* value noted<br>*Give P values as exact values whenever suitable.*
☒	☐	For Bayesian analysis, information on the choice of priors and Markov chain Monte Carlo settings
☒	☐	For hierarchical and complex designs, identification of the appropriate level for tests and full reporting of outcomes
☒	☐	Estimates of effect sizes (e.g. Cohen's *d*, Pearson's *r*), indicating how they were calculated

Our web collection on statistics for biologists contains articles on many of the points above.

Software and code

Policy information about availability of computer code

Data collection	Image acquisition softwares used were: xcellence (Olympus), Softworx (Applied precision). Mass spectrometry acquisition: Xcalibur (Thermo Scientific), MO.Control (Nanotemper), GeneSys (SYNGENE)
Data analysis	Custom ImageJ macros were used to determine speed and runlength (Available on github.com/cmcb-warwick). Figures were prepared by adjusting min/max and inverting look up tables using ImageJ and assembled using Adobe Illustrator, Statistical data analyses and graphs were generated using Origin Pro 8.5 (OriginLab), python's Matplotlib and Scipy packages or R.

For manuscripts utilizing custom algorithms or software that are central to the research but not yet described in published literature, software must be made available to editors/reviewers. We strongly encourage code deposition in a community repository (e.g. GitHub). See the Nature Research guidelines for submitting code & software for further information.

Data

Policy information about availability of data

All manuscripts must include a data availability statement. This statement should provide the following information, where applicable:

- Accession codes, unique identifiers, or web links for publicly available datasets
- A list of figures that have associated raw data
- A description of any restrictions on data availability

Our crosslink mass spec data are available via ProteomeXchange with identifier PXD013939. We provide our BioID mass spec data in supplementary data 1. Other raw data are in the source data file or available upon request.

Field-specific reporting

Please select the one below that is the best fit for your research. If you are not sure, read the appropriate sections before making your selection.

☒ Life sciences ☐ Behavioural & social sciences ☐ Ecological, evolutionary & environmental sciences

For a reference copy of the document with all sections, see nature.com/documents/nr-reporting-summary-flat.pdf

Life sciences study design

All studies must disclose on these points even when the disclosure is negative.

Sample size	Sample size was determined from individual experiments carried out.
Data exclusions	We did not exclude outlier data points from experiments or experimental repeats with exception of XL-MS experimental runs with low protein coverage.
Replication	Replications were independent experiments unless otherwise indicated in the methods.
Randomization	No randomization was done.
Blinding	The landing rates and frequency of running motors for KIF1C, PTPN21FERM and EzrinFerm data sets were verified by a blinded data analyst.

Reporting for specific materials, systems and methods

We require information from authors about some types of materials, experimental systems and methods used in many studies. Here, indicate whether each material, system or method listed is relevant to your study. If you are not sure if a list item applies to your research, read the appropriate section before selecting a response.

Materials & experimental systems

n/a	Involved in the study	
☐	☒	Antibodies
☐	☒	Eukaryotic cell lines
☒	☐	Palaeontology
☒	☐	Animals and other organisms
☒	☐	Human research participants
☒	☐	Clinical data

Methods

n/a	Involved in the study	
☒	☐	ChIP-seq
☒	☐	Flow cytometry
☒	☐	MRI-based neuroimaging

Antibodies

Antibodies used	anti-cortactin clone 4F11, (Cat #05-180, Lot #2290226, Millipore)<br>anti-mouse IgG 647 conjugate (Cat #A31571, Lot #47735A Molecular probes)<br>anti-KIF1C (Cat #AKIN11, Lot #011, Cytoskeleton)<br>anti-rabbit IgG HRP conjugate secondary (Cat#W401B, Lot #237671, Promega),<br>anti-Flag (Cat #F3165, Lot #058K6113, Sigma),<br>anti-HA (C29F4, #3724S, Lot #9, CST),<br>anti-Hook3 (#15457-1-AP, Proteintech),
Validation	Antibodies were verified by the manufacturer. In addition, we validated anti-KIF1C in Theisen et al., Dev Cell, 2012. The anti-cortactin antibody was validated as podosome marker in Efimova et al, J Cell Science 2014.

Eukaryotic cell lines

Policy information about cell lines

Cell line source(s)	hTERT RPE1 cells (Clontech) were used to establish the RPE1 α5-integrin-GFP stable cell line described here.<br>A7r5 (ATCC), HEK293 (Agilent), SF9 (VWR)
Authentication	RPE1, HEK293 and SF9 cell lines were not authenticated. A7r5 cell lines were authenticated for Transgelin, a smooth muscle specific marker.
Mycoplasma contamination	Mycoplasma testing is carried out monthly. Experiments are only performed on mycoplasma negative cell lines.

Commonly misidentified lines (See ICLAC register)	The cell lines used in this study are not mentioned in the misidentified ICLAC register
